# Exogenous Zeaxanthin Alleviates Low Temperature Combined with Low Light Induced Photosynthesis Inhibition and Oxidative Stress in Pepper (*Capsicum annuum* L.) Plants

**DOI:** 10.3390/cimb44060168

**Published:** 2022-05-25

**Authors:** Dongxia Ding, Jing Li, Jianming Xie, Nenghui Li, Emily Patience Bakpa, Kangning Han, Yan Yang, Cheng Wang

**Affiliations:** College of Horticulture, Gansu Agricultural University, Lanzhou 730070, China; dingdongxia100741@outlook.com (D.D.); lj@gsau.edu.cn (J.L.); lnh9656@outlook.com (N.L.); emilybakpa@gmail.com (E.P.B.); h18893914723@163.com (K.H.); yangyan1061@outlook.com (Y.Y.); gsauphd0810@outlook.com (C.W.)

**Keywords:** pepper, zeaxanthin, low temperature combined with low light, ascorbate–glutathione cycle, oxidative stress

## Abstract

Low temperature combined with low light (LL) affects crop production, especially the yield and quality of peppers, in northwest China during the winter and spring seasons. Zeaxanthin (Z) is a known lipid protectant and active oxygen scavenger. However, whether exogenous Z can mitigate LL-induced inhibition of photosynthesis and oxidative stress in peppers remains unclear. In this study, we investigated the effects of exogenous Z on photosynthesis and the antioxidant machinery of pepper seedlings subject to LL stress. The results showed that the growth and photosynthesis of pepper seedlings were significantly inhibited by LL stress. In addition, the antioxidant machinery was disturbed by the uneven production and elimination of reactive oxygen species (ROS), which resulted in damage to the pepper. For example, membrane lipid peroxidation increased ROS content, and so on. However, exogenous application of Z before LL stress significantly increased the plant height, stem diameter, net photosynthetic rate (Pn), and stomata, which were obviously closed at LL. The activities of antioxidant enzymes superoxide dismutase (SOD), catalase (CAT), mono de-hydroascorbate reductase (MDHAR), de-hydroascorbate reductase (DHAR), ascorbate peroxidase (APX), and ascorbate oxidase (AAO) improved significantly due to the increased expression of *CaSOD*, *CaCAT*, *CaAPX*, *CaMDHAR*, and *CaDHAR*. The ascorbic (AsA) and glutathione (GSH) contents and ascorbic/dehydroascorbate (AsA/DHA) and glutathione/oxidized glutathione (GSH/GSSG) ratios also increased significantly, resulting in the effective removal of hydrogen peroxide (H_2_O_2_) and superoxide anions (O_2_^•−^) caused by LL stress. Thus, pre-treatment with Z significantly reduced ROS accumulation in pepper seedlings under LL stress by enhancing the activity of antioxidant enzymes and accumulation of components of the ascorbate–glutathione (AsA–GSH) cycle and upregulated key genes in the AsA–GSH cycle.

## 1. Introduction

Temperature and light are two of the most fundamental and important signals that regulate plant growth and development; they directly provide the energy required for growth and facilitate the spatiotemporal development of plants [1,2]. The pepper plant, *Capsocum annuum* (L.), which possesses both thermophilic and heliophilic characteristics, is one of the most important vegetable crops grown off-season in greenhouses in north-western China. However, pepper cultivation is often affected by low temperatures combined with low light (LL) conditions during autumn-winter and winter-spring, which limits their growth and development [3,4,5]; the LL environment leads to a decrease in crop yield that affects the economic viability of pepper [6].

Plant photosynthesis is affected by abiotic stresses such as low temperature, low light, or low temperature combined with low light. Hu et al. (2017) [7] found that low light inhibits photosynthetic electron transport and carbon assimilation enzyme activity in the Chinese mini cabbage (*Brassica pekinensis* L.). According to a study by Ma et al. (2018) [8], low temperatures reduce membrane fluidity, cause low water availability, and an irreversible reduction in photosynthetic rate. Low-temperature stress can also lead to reduced photosynthetic efficiency in perennial ryegrass [9]. LL stress in cucumber has led to lipid peroxidation of membraned, downregulation of Rubisco gene expression, reduction of biomass, and photosynthetic capacity. LL resulted in a large number of reactive oxygen species (ROS) in plant organelles, which mainly include superoxide anions, hydrogen peroxide, and hydroxyl free radicals [10]. Severe stress conditions can cause oxidative damage to cellular components, lipid and protein peroxidation, DNA fragmentation, enzyme inhibition, and the activation of programmed cell death pathways, eventually leading to plant death [11,12,13]. Previous studies have shown that low-temperature stress in peppers leads to increased ROS generation and a decreased antioxidant enzyme activity [14]; stems and leaves of pepper plants appeared weak after 24 h of low-temperature stress at 8 °C due to induced oxidative stress [15]. Plants have developed several strategies to detect and eliminate excess ROS and alleviate the damage caused by the excessive accumulation of ROS in plant cells. The antioxidant system in plants includes both enzymatic and non-enzymatic defense systems [16,17]. The antioxidant enzyme system consists primarily of superoxide dismutase (SOD), peroxidase (POD), and catalase (CAT), which mitigate oxidative damage to plants under stress [18]. The ascorbate–glutathione cycle (AsA–GSH) is also an important pathway for the ROS scavenging in plants; in the AsA–GSH cycle, ascorbate peroxidase (APX) is involved in the removal of hydrogen peroxide from the cytoplasm and chloroplasts [19]. Non-enzymatic defense systems in plants mainly include ascorbic acid (AsA), glutathione (GSH), proline, and flavonoids, which serve as substrates for enzymes or directly act as ROS scavengers. Reduced AsA and GSH are important antioxidant substances in the AsA–GSH cycle that can directly scavenge ROS and serve as enzyme substrates to scavenge ROS [20,21]. Ascorbate oxidase (AAO), monodehydroascorbate acid reductase (MDHAR), dehydroascorbate acid reductase (DHAR), and APX are the major enzymes involved in antioxidant regeneration and ROS scavenging in plant cells [22,23].

Zeaxanthin (Z) is a carotenoid found mainly in thylakoids—also in the chloroplast envelope—where it is involved in photoprotection via a role in thermal energy dissipation and antioxidation [24]. Z content in peppers, when increased under low temperatures and low light, showed improved stress tolerance [25]. As an antioxidant in plants, Z acts as a lipid protectant and ROS scavenger [26]; Z directly quenches ROS [27] and triggers non-photochemical quenching of triplet chlorophyll and singlet oxygen—thereby playing a protective role in the photosynthetic system of plant cells [28,29]. Studies have shown that overexpression of the tomato (*Lycopersicum esculentum* Mill.) zeaxanthin epoxidase (*LeZE*) has resulted in increased Z content and high abiotic stress tolerance [30,31]. The results obtained by Chen et al. (2018) [32] showed that overexpression of the lycopene-β-cyclase gene (*IBLCYB*2) significantly increased the Z content in transgenic sweet potatoes and also significantly improved the tolerance of the transgenic plants to oxidative stress. The presence of Z in thylakoids increases their tolerance to lipid peroxidation in plants [33]. In addition, free Z is found in thylakoid lipids and scavenges singlet oxygen produced during photosynthesis [34]. Compared to wild-type tobacco, overexpression of alfalfa zeaxanthin cycidase (*MsZEP*) has shown increased tolerance to low-light stress [35]. The above studies show that Z plays an important role in plant response to stress.

In the present study, we analyzed the effects of exogenous Z on the antioxidant system, glutathione circulation system, and relative expression levels of antioxidant enzymes in pepper plants under LL stress. The results of these experiments would help improve the understanding of LL tolerance mechanisms in pepper and contribute to the development of strategies to mitigate LL challenges in pepper cultivation.

## 2. Materials and Methods

### 2.1. Plant Material and Growth Conditions

Pepper seeds of “Hangjiao No. 2” obtained from Tianshui Shenzhou Lvpeng Agricultural Technology Company (Tianshui, China). Seeds were soaked in water with stirring for 30 min at 55 °C and soaked for a further 6 h in water at 25 °C. The seeds were then placed in the dark in an artificial climate box at 28 °C and 75% humidity to accelerate germination. Two seeds with approximately 1 mm radicle were sown in a nutrient bowl (9 cm × 9 cm) filled with a substrate (peat: vermiculite: perlite = 3:1:1) to establish seedlings. In the climate chamber, conditions of 28 °C 300 μmol m^−2^ s^−1^/18 °C 0 μmol m^−2^ s^−1^ (day/night) at 70% humidity were maintained, and all seedlings were kept uniform.

### 2.2. Treatments

When the sixth leaf of the seedlings was fully developed (about 45 days), seedlings with uniform growth were selected and divided into four treatment groups. Per treatment contained 50 pepper seedlings. (1) Normal treatment (CK, 28 °C/18 °C, day/night, 300 μmol m^−2^ s^−1^), (2) normal treatment + zeaxanthin (CK + Z, 28 °C/18 °C, day/night, 300 μmol m^−2^ s^−1^, 50 mg·L^−1^ of zeaxanthin), (3) low temperature combined with low light (LL, 15 °C/5 °C, day/night, 100 μmol m^−2^ s^−1^), (4) low temperature combined with low light + zeaxanthin (LL + Z, 15 °C/5 °C, day/night, 100 μmol m^−2^ s^−1^, 50 mg·L^−1^ zeaxanthin). The leaves of CK + Z and LL + Z were sprayed with 50 mg·L^−1^ zeaxanthin, and those of CK and LL were both sprayed with the same volume of distilled water, which served as all control. The foliar sprays included 0.01% Tween 80; the leaves of the seedlings were sprayed at 20:00 on both sides without dripping for 4 consecutive days. After pretreatments, seedlings were placed in artificial climate chambers for the treatments and growth a relative humidity of 70–80% and 12 h photoperiod. Five seedlings were randomly selected from each treatment at 0, 6, 12, 24, 48, and 168 h; functional leaves were collected from each of the seedlings to determine relevant indices.

### 2.3. Biomass and Morphology

After 168 h of treatment, five seedlings from each treatment were randomly selected to measure growth indices, including plant height, stem diameter, and dry and fresh weight measurements. Plant height was measured using a tape measure as the distance from the base of the pepper stem to the growing point. Stem diameter was measured using a Vernier caliper. The pepper seedlings were then cleaned with ultrapure water and divided into roots and shoots. After measuring the fresh weight, the seedlings were dried at 105 °C for 15 min and then at 80 °C, after which dry weight measurements were recorded.

### 2.4. Measurement of Electrolyte Leakage and Relative Water Content

Electrolyte leakage (EL) was measured using the third functional leaf with an electrical conductivity meter (DDSJ-308A, Shanghai, China) according to the method of Min (2019) [36]. The third leaf from each seedling of each treatment was washed with ultrapure water. Twelve leaf discs (1 cm) were then prepared using a hole punch. The leaf discs were immersed in 15 mL of deionized water, vacuumed for 30 min, and shaken for 3 h (L1). The electrical conductivity of deionized water is L0. Finally, the electrical conductivity (L2) was determined after being heated in boiling water for 10 min and cooled. The EL was calculated using the following formula: EL % = (L1 − L0)/(L2 − L0) × 100.

The relative water content of leaves was determined using the method described by Min et al. (2019) [36].

### 2.5. Scanning Electron Microscopy

After 24 and 48 h treatment periods, leaves were collected in three replicates to observe the stomata morphology under each treatment using a scanning electron microscope (SEM, HITACHI-S3400N). After 24 and 48 h, according to the method described by Min (2019) with some modifications [36]. Leaves were cut into 5 mm × 5 mm segments on both sides of the main vein and soaked in a 4% glutaraldehyde fixing solution for 2 h at 25 °C. The leaves were then washed four times at 10 min intervals with phosphoric acid buffer (0.10 M PBS, pH 6.8). The leaf samples were then dehydrated with increasing concentrations of ethanol (30, 40, 50, 60, 70, and 75%), 20 min per concentration, and finally soaked in 75% ethanol about 12 h. On the second day, the samples were dehydrated with ethanol at different concentration gradients (75, 80, 85, 90, and 95%) for 20 min each and then transferred to 100% ethanol three times for 30 min each. Finally, ethanol was eluted out with a series of tert-butyl alcohol samples of increasing concentrations (30, 50, 70, 80, 85, 90, 95, and 100%) for 30 min each. The samples were then dried; each sample was then sprayed with a thin layer of gold for observation under SEM.

### 2.6. Determination of Photosynthetic Gas Exchange Parameters

Photosynthetic parameters, including net photosynthetic rate (Pn), intercellular CO_2_ concentration (Ci), transpiration rate (Tr), and stomatal conductance (Gs), were measured 168 h after treatment of fully expanded, functional leaves from 8:30 A.M. to 11 A.M. using the CIRAS-2 Portable Photosynthesis System (PP system, Amesbury, UK). Photosynthetic apparatus parameters were set to a CO_2_ concentration: 380 μmol mol^−1^, light intensity: 1000 μmol m^−2^ s^−1^, temperature: 25 °C, and airflow rate: 120 L h^−1^. Data were recorded after the values were stable [25,37].

### 2.7. Determination of Superoxide Anion Content and Tissue Nitroblue Tetrazolium (NBT) Staining

The superoxide anion content was determined using an assay kit from Suzhou Keming Biotechnology Co., (Suzhou, China). The leaves were stained with NBT [38]. Leaves of pepper seedlings from the different treatments were placed in 150 mL wide-mouth triangular flasks, and 50 mL of NBT dye solution (1 mg/mL, dissolved in PBS at pH = 7.8) was added and then vacuumed for 30 min. They were then incubated in the dark at 25 °C for 6 h. The dye solution was discarded, and a decolorization solution (lactic acid:glycerol:anhydrous ethanol = 1:1:3 (volume ratio) was added to the flasks, which were placed in a boiling water bath for 5–6 min until the green color of the leaves faded completely. The NBT stained leaves were scanned using a scanner (WinRHIZO Pro LA2400, Regent Instruments Inc., Quebec, QC, Canada).

### 2.8. Determination of Hydrogen Peroxide Content and Tissue Diaminobenzene (DAB) Staining

Hydrogen peroxide content was determined using an assay kit from Suzhou Keming Biological Technology Co, Ltd., (Suzhou, China). DAB staining of leaves was performed according to the method described in Ma (2013) [38]. Leaves from different treatment groups were placed in a wide-necked triangular flask (150 mL), to which 50 mL of DAB dye solution (1 mg/mL, pH = 7.0) was then added and vacuumed for 1 h. Then, the leaves were incubated overnight in the dark at 25 °C. Decolorization and scanning procedures were followed as per the NBT method.

### 2.9. Determination of Antioxidant Enzyme Activity

The enzyme activities of SOD (EC1.15.1.1), POD (EC1.11.1.7), CAT (EC1.11.1.6), AAO (EC1.10.3.3), DHAR(EC1.8.5.1), MDHAR (EC1.6.5.4), and APX (EC1.11.1.11) were determined using the kit obtained from Suzhou Keming Biological Technology Co., Ltd. The specific steps were determined according to the kit instructions. Briefly, the SOD activity was estimated by measuring its ability to inhibit the reduction of WST-8, and the absorbance of the formazan solution (reduction product of WST-8) was read at 450 nm. The CAT activity was measured by the absorption value of H_2_O_2_ decreasing with time extension at 240 nm. The POD activity was measured by the increased absorbance value at 470 nm after guaiacol was oxidized by H_2_O_2_. AAO can oxidize AsA directly, and the activity of AAO can be calculated by measuring the oxidation amount of AsA. MDHAR catalyzes the reduction of mono-dehydroascorbic acid (MDHA) by nicotinamide adenine dinucleotide (NADH), and the activity of MDHAR is calculated by the absorption value of NADH at 340 nm. DHAR enzyme catalyzes reduction of DHA by GSH to generate AsA. DHAR activity can be calculated by measuring the reduction rate of DHA. APX catalyzes the oxidation of AsA by hydrogen peroxide, and the activity of APX is calculated by measuring the oxidation rate of AsA.

### 2.10. Determination of AsA, DHA, GSH, and GSSG Contents

The AsA, DHA, GSH, and GSSG contents were measured using reagent kits (Suzhou Keming Biological Technology Co., Ltd., Suzhou, China) according to the manufacturer’s instructions. In order to determine of AsA, ascorbic acid was treated with Fast Blue B salt to produce a yellow oxalohydrazine-2-hydroxybutanoyllactone derivative, and the absorbance was measured at 420 nm in an acetic acid solution. DHA was measured indirectly using dithiothreitol, which reduced DHA to AsA, and DHA was measured using the rate of formation of AsA. For the determination of GSH, 2-nitro-5-mercaptobenzoic acid, a yellow chemical, was prepared by treating GSH with 5,5-dithiobis- and 2-nitrobenzoic acid. GSH was determined by measuring the resultant product at the maximum absorption wavelength of 412 nm. For the determination of GSSG, 2-vinylpyridine was used to inhibit intrinsic GSH in the cell, after which GSSG was reduced by glutathione reductase and measured using the GSH method.

### 2.11. Extraction of Total RNA and RT-PCR Analysis

Total RNA was extracted using an RNA extraction kit (Tiangen Biotech Co., Ltd., Beijing, China) according to the manufacturer’s instructions. The extracted total RNA was used to synthesize first-strand cDNA using a cDNA kit (Tiangen Biotech Co., Ltd., Beijing, China). The gene sequence was obtained from the NCBI website (https://www.ncbi.nlm.nih.gov/gene (accessed on 2 August 2021)). Primers were synthesized by Shanghai Biotechnology Co., Ltd. The primer sequences used are listed in Table 1. Actin was used as the internal reference. qRT-PCR was performed using an ABI 7500 real-time PCR system. The reaction mixture (20 μL) contained 2 μL cDNA solution (100 ng/μL), 0.4 μL forward primer, 0.4 μL reverse primer, 10 μL 2*TransStart^®^ Tip Green qPCR SuperMix, and 7.2 μL nuclease-free water. Reaction conditions were as follows: 94 °C for 30 s, followed by 40 cycles of 94 °C for 5 s and 60 °C for 30 s. Three PCR replicates were performed per sample, and the 2^−^^△△ct^ method was used to calculate relative expression levels. Three biological replicates were used for total RNA extraction, and three technical replicates were used for qPCR.

### 2.12. Statistical Analysis

The experiments for each treatment were repeated three times, except for the morphological characteristics analysis, which was repeated five times. Results were analyzed by one-way analysis ANOVA, and then Duncan’s multiple range test was performed to detect a significant difference at *p* < 0.05. All images were drawn using Origin Pro 9.0 (Origin Lab Institute Inc., Northampton, MA, USA). Photoshop CS6 software (Adobe, San Jose, CA, USA) was used for image processing.

## 3. Results

### 3.1. Effects of Exogenous Zeaxanthin on the Morphological Characteristics of Pepper under LL Stress

No differences were observed in morphological traits such as plant height and stem diameter of pepper seedlings, with exogenous Z application under normal growing conditions (Table 2). However, compared to CK, there was a significantly reduced in plant height, stem diameter, dry weight, fresh weight of shoots, and dry weight and fresh weight of roots of pepper seedlings under LL stress. Compared to LL, each growth index of pepper seedlings was increased under the LL + Z treatment; plant height, stem diameter, and shoot dry weight increased by 11.70%, 13.67%, and 16.60%, respectively.

**Figure 1:**
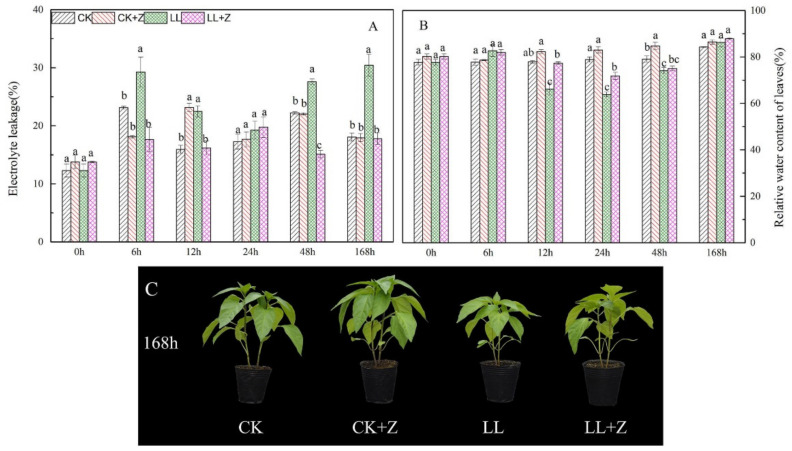
Effects of exogenous zeaxanthin on electrolyte leakage, relative water content and growth of pepper leaves under low temperature combined with low-light stress. (**A**): electrolyte leakage; (**B**): relative water content; (**C**): growth of pepper. CK: the pepper was sprayed with distilled water under normal conditions, CK + Z: the pepper was sprayed with zeaxanthin under normal conditions, LL: the pepper was sprayed with distilled water under low temperature combined with low-light stress, and LL + Z: the pepper was sprayed with zeaxanthin under low temperature combined with low-light stress. The values were presented as means ± standard deviations (SDs; *n* = 3). The different letters above bars indicated significant difference at *p* < 0.05 in the same period among treatments.

Pepper seedlings from each treatment were photographed and observed at 168 h after treatment (Figure 1C). The results showed that the height of pepper plants under LL stress was significantly lower than that under CK, and their growth was weaker. The LL + Z treatment was better than the LL treatment in terms of seedling growth, which had a mitigating effect on plant growth inhibition caused by low temperature and low light.

### 3.2. Electrolyte Leakage and Relative Water Content

Pepper seedlings under LL stress showed a significant increase in EL at all observed time points, except at 24 h compared to that under normal treatment (Figure 1A). Moreover, EL showed an initial increasing trend with increasing processing time, followed by a decreasing trend, and then increased again, with an increase of 26.62%, 41.00%, 11.41%, 24.21%, and 68.65% during 6–168 h, respectively. However, seedlings under LL + Z showed a significant reduction in EL compared to that under LL treatment, except at 24 h. Pre-treatment with Z reduced the EL of pepper seedlings, improved cell membrane stability, and reduced the damage caused by LL stress in pepper seedlings. Compared to that of CK, the RWC of leaves decreased under LL stress—reached the lowest point until 24 h of stress—and then gradually increased; at 168 h, the RWC appeared to have no significant difference in all treatments (Figure 1B). After 12 h, 24 h, and 48 h of LL stress treatment, the leaf RWC decreased by 15.04%, 19.08%, and 6.24%, respectively, compared with that of CK. However, the application of Z significantly increased leaf RWC by 16.87% and 12.53% after 12 and 24 h, respectively, compared with that of LL seedlings. Under normal growth conditions, no significant differences were observed in EL and RWC in pepper seedlings with or without Z pre-treatment.

### 3.3. Microscopic Structure of Leaf Tissue Stress

**Figure 2:**
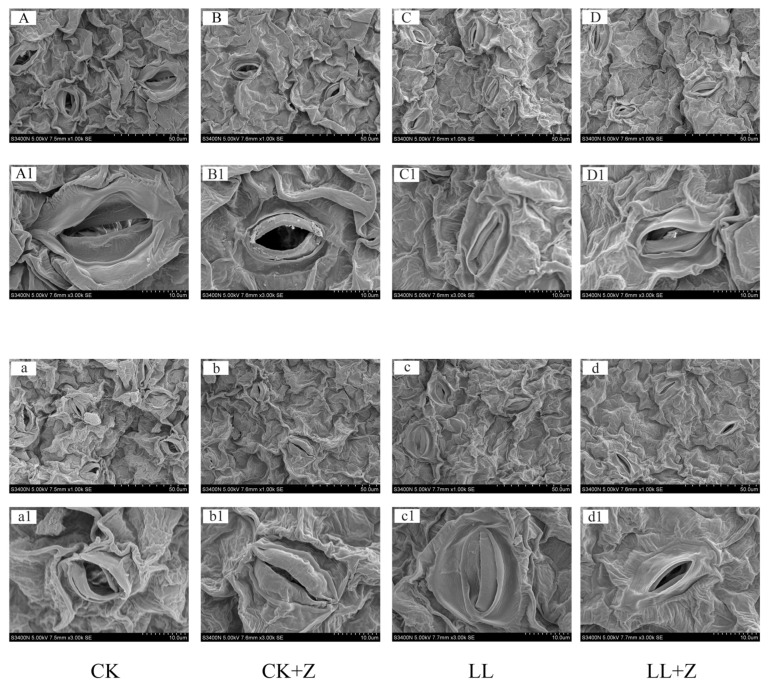
Effects of exogenous zeaxanthin on stomatal morphology of pepper leaves under low temperature combined with low-light stress. CK: the pepper was sprayed with distilled water under normal conditions, CK + Z: the pepper was sprayed with zeaxanthin under normal conditions, LL: the pepper was sprayed with distilled water under low temperature combined with low-light stress, and LL + Z: the pepper was sprayed with zeaxanthin under low temperature combined with low-light stress. (**A**–**D**) ×1000 and (**A1**–**D1**) ×3000 represent stomatal morphology of pepper leaves after 24 h treatment, and (**a**–**d**) ×1000 and (**a1**–**d1**) ×3000 represent stomatal morphology of pepper leaves after 48 h treatment.

Stomata are gateways for gas exchange between plant leaves and the external environment. Stomata characteristics may reflect the response of plants to environmental changes. Figure 2 shows that under LL stress, guard cells of pepper seedlings lost water which caused the shriveling of stomata. The shape of the stomata was deformed after 24 h of stress, and after 48 h, the shape of the stomata returned to normal but remained closed. However, under the LL + Z treatment, the guard cells recovered from water loss, and most of the stomata were half-open, indicating that Z pre-treatment could cause the stomata to open.

### 3.4. Effects of Exogenous Zeaxanthin on the Photosynthetic Parameters of Pepper under LL Stress

LL stress significantly reduced Pn (98.11%), Tr (64.65%), and Gs (64.37%), whereas Ci increased by 37.84% when compared with that of CK (Table 3). However, the Pn of pepper leaves significantly increased in the LL + Z treatment when compared to that without LL pre-treatment, whereas Ci showed an opposite trend.

### 3.5. Effects of Exogenous Zeaxanthin on Reactive Oxygen Content in Pepper Leaves under LL Stress

**Figure 3:**
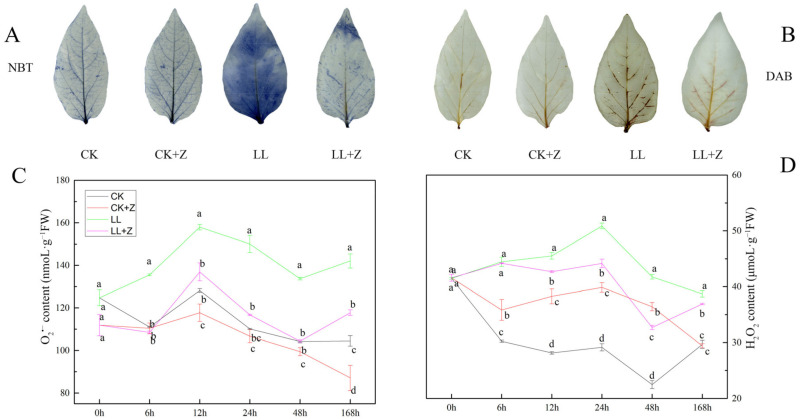
Effects of exogenous zeaxanthin on hydrogen peroxide and superoxide anion in pepper leaves under low temperature combined with low-light stress. (**A**) NBT staining (168 h); (**B**) DAB staining (168 h); (**C**) O_2_^•−^ content; (**D**) H_2_O_2_ content. CK: the pepper was sprayed with distilled water under normal conditions, CK + Z: the pepper was sprayed with zeaxanthin under normal conditions, LL: the pepper was sprayed with distilled water under low temperature combined with low-light stress, and LL + Z: the pepper was sprayed with zeaxanthin under low temperature combined with low-light stress. The values were presented as means ± standard deviations (SDs; *n* = 3). The different letters represent significant difference at *p* < 0.05 in the same period among treatments.

The content of hydrogen peroxide (H_2_O_2_) and superoxide ion (O_2_^•−^) in pepper leaves was qualitatively analyzed after 168 h by NBT and DAB staining (Figure 3A,B). Under LL stress, the staining of NBT and DAB was deeper than that of CK, which indicated an increase in ROS after LL stress treatment. However, under LL stress, NBT and DAB were brighter than CK in the LL + Z treatment, indicating that Z could remove excess ROS accumulated under LL stress to some extent.

Quantitative analysis of H_2_O_2_ and O_2_^•−^ in the pepper leaves of each treatment group—showed that in the case of LL +Z, the changes in O_2_^•−^ content coincided with the changes in H_2_O_2_ content—which was significantly lower than that under LL stress (Figure 3C,D). Compared with LL treatment, the O_2_^•−^ content in LL + Z treatment decreased by 13.31%, 22.19%, 21.97%, and 17.12% during 12–168 h, respectively; the H_2_O_2_ content decreased by 6.23%, 13.18%, 21.74%, and 4.62% during 12–168 h, respectively. Pre-treatment with Z under normal conditions could significantly reduce the O_2_^•−^ content after 168 h, but this treatment did not affect the H_2_O_2_ content.

### 3.6. Effects of Exogenous Zeaxanthin on Activities of SOD, POD, and CAT Enzymes in Pepper under LL Stress

**Figure 4:**
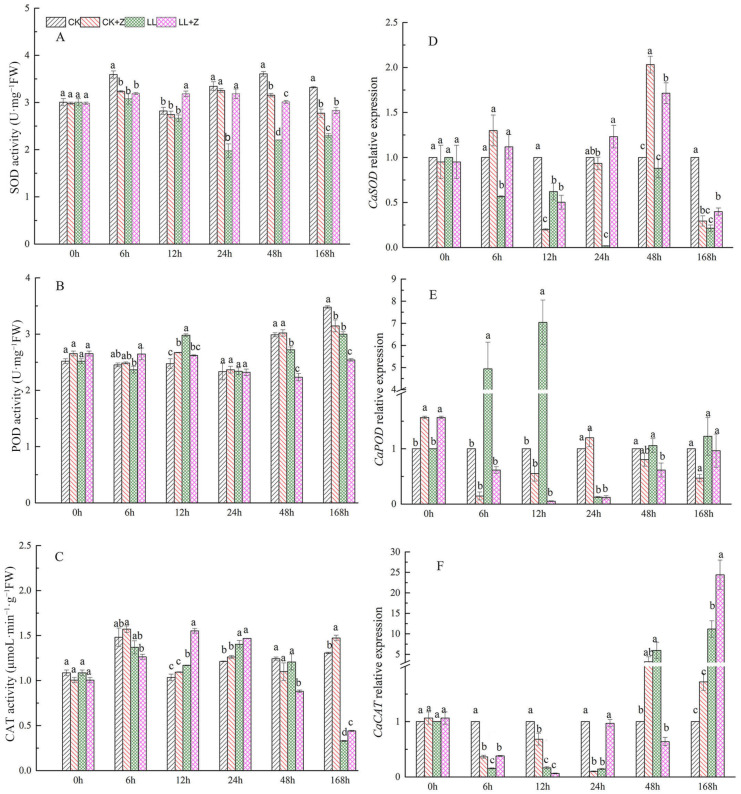
Effects of exogenous zeaxanthin on antioxidant enzyme activities and related gene expression in pepper leaves under low temperature combined with low-light stress. (**A**) SOD activity; (**B**) POD activity; (**C**) CAT activity; (**D**) *CaSOD* relative expression; (**E**) *CaPOD* relative expression (**F**) *CaCAT* relative expression. CK: the pepper was sprayed with distilled water under normal conditions, CK + Z: the pepper was sprayed with zeaxanthin under normal conditions, LL: the pepper was sprayed with distilled water under low temperature combined with low-light stress, and LL + Z: the pepper was sprayed with zeaxanthin under low temperature combined with low-light stress. The values were presented as means ± standard deviations (SDs; *n* = 3). The different letters above bars indicated significant difference at *p* < 0.05 in the same period among treatments.

SOD activity slightly decreased after Z pretreatment under normal conditions when compared with that in CK (Figure 4A). Seedlings under LL stress decreased dramatically—reaching the lowest point after 24 h—with a significant decrease of 40.94%. The exogenous application of Z increased the SOD activity of pepper seedlings by 19.39%, 61.23%, 36.62%, and 22.96% over the period of 12–168 h when compared to that under LL stress without pre-treatment.

Under LL conditions, the activity of CAT in the leaves increased significantly after 12 h and 24 h. Thereafter, it decreased significantly with increasing duration of stress and decreased by 74.82% in pepper leaves when compared with the CK after 168 h (Figure 4C). After 12 h and 168 h of LL stress, LL + Z seedlings showed an increase in CAT activity by 32.73% and 34.12%, respectively.

The results displayed in Figure 4B show that the activity of POD in pepper leaves first increased under LL stress and then decreased compared to that in CK seedlings. After 12 h of LL stress, the activity of POD increased by 20.49% but decreased by 8.92% and 13.79% after 48 and 168 h, respectively. The activity of POD initially increased under LL + Z treatment and then decreased under stress, decreasing by 12.08%, 17.89%, and 15.33% after 12 h, 48 h, and 168 h, respectively, when compared to that under LL stress.

The transcription of three genes related to the antioxidant system (SOD, CAT, and POD) were significantly activated in LL + Z seedlings (Figure 4D–F). Except at 12 h, the *CaSOD* expression level in LL + Z seedlings was significantly higher than under LL stress without pre-treatment. In the early stages of LL stress treatment, the expression of the *CaCAT* gene was significantly downregulated, but it showed an increase in expression leaves with time. After 168 h, the *CaCAT* gene was 11.16 times higher in the LL treatment than that in the CK treatment and 2.19 times higher in the LL + Z treatment than that in the LL stress. Under LL stress, the relative expression of the *CaPOD* gene was significantly upregulated by 4.94- and 7.04-fold compared to CK after 6 and 12 h, respectively, but *CaPOD* gene expression was downregulated in the case of the Z pretreated seedling.

### 3.7. Effects of Exogenous Zeaxanthin on Ascorbic Acid Content in Pepper under LL Stress

**Figure 5:**
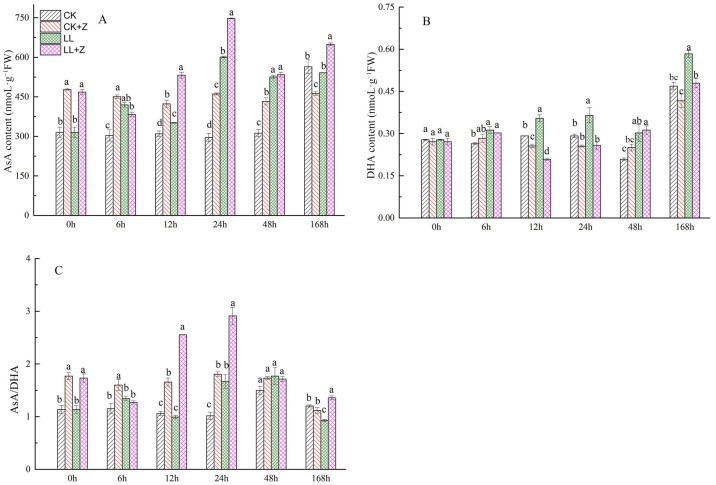
Effects of exogenous zeaxanthin on AsA and DHA contents and AsA/DHA ratio in pepper leaves under low temperature combined with low-light stress. (**A**) AsA content; (**B**) DHA content; (**C**) AsA/DHA ratio. The values were presented as means ± standard deviations (SDs; *n* = 3). CK: the pepper was sprayed with distilled water under normal conditions, CK + Z: the pepper was sprayed with zeaxanthin under normal conditions, LL: the pepper was sprayed with distilled water under low temperature combined with low-light stress, and LL + Z: the pepper was sprayed with zeaxanthin under low temperature combined with low-light stress. The different letters above bars indicated significant difference at *p* < 0.05 in the same period among treatments.

The AsA and DHA contents in the leaves of pepper seedlings increased significantly under LL stress when compared with that under CK, but the change was no longer significant after 168 h of stress (Figure 5A,B). The DHA and AsA contents increased by 18.42%, 21.43%, 25.05%, 45.00% and 38.20%, 13.32%, 103.33%, 68.31%, respectively, compared to that of CK during 6–48 h of LL stress. In addition, LL + Z seedlings significantly increased AsA content at 12 h, 24 h, and 168 h compared to that of LL; however, DHA content decreased by 41.18%, 29.17%, and 17.86% at these three stress time points.

The AsA/DHA ratio in LL treated pepper seedlings increased by 64.16% at 24 h, but decreased by 22.76% at 168 h with the extension of stress duration when compared with that in CK seedlings (Figure 5C). The ratio of AsA/DHA in the leaves of LL + Z seedlings increased by 157.09%, 74.69%, and 46.15% at 12 h, 24 h, and 168 h, respectively, when compared to that of the LL stress seedlings. These results indicate that Z can effectively increase antioxidant content and improve the antioxidant capacity of pepper seedlings under LL stress.

### 3.8. Effects of Exogenous Zeaxanthin on Glutathione Content in Pepper under LL Stress

**Figure 6:**
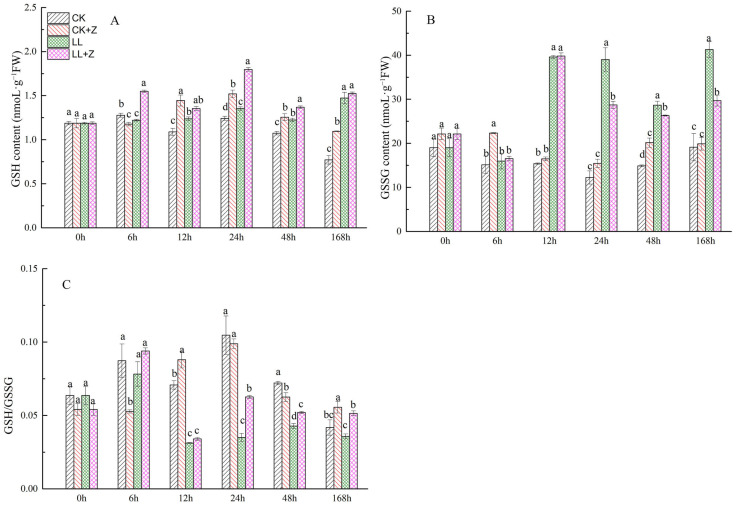
Effects of exogenous zeaxanthin on GSH and GSSG contents and GSH/GSSG ratio in pepper leaves under low temperature combined with low-light stress. (**A**) GSH content; (**B**) GSSG content; (**C**) GSH/GSSG ratio. CK: the pepper was sprayed with distilled water under normal conditions, CK + Z: the pepper was sprayed with zeaxanthin under normal conditions, LL: the pepper was sprayed with distilled water under low temperature combined with low-light stress, and LL + Z: the pepper was sprayed with zeaxanthin under low temperature combined with low-light stress. The values were presented as means ± standard deviations (SDs; *n* = 3). The different letters above bars indicated significant difference at *p* < 0.05 in the same period among treatments.

Figure 6A shows that GSH content in the leaves of pepper seedlings first decreased and then increased under LL stress; after 6 h of stress, GSH content decreased by 4.39%. Subsequently, GSH content increased significantly with increasing stress duration by 13.72%, 23.40%, 14.01%, and 90.99% at 12 h–168 h, respectively. Pre-treatment with Z further promoted the increase in GSH content in leaves under LL stress, which increased by 26.89%, 32.81%, and 11.85% after 6 h, 24 h, and 48 h, respectively. Compared to the normal treatment, the GSSG content greatly increased under LL stress, but no difference was observed at 6 h of LL stress (Figure 6B). Under LL + Z, there was a significant reduction in the GSSG content of pepper seedlings by 26.42%, 8.22%, and 28.09% after 24 h, 48 h, and 168 h, respectively, when compared with that of LL seedlings. The ratio of GSH/GSSG in leaves under LL stress decreased by 55.89%, 66.53%, and 40.63% after 12 h, 24 h, and 48 h, respectively, when compared with that of CK seedlings. In contrast, LL + Z seedlings showed a significant improvement in the GSH/GSSG ratio by 78.75%, 21.59%, and 43.98% after 24 h, 48 h, and 168 h, respectively, compared with that of LL stress (Figure 6C).

### 3.9. Effects of Exogenous Zeaxanthin on the Activities of Ascorbate Oxidase and Ascorbate Peroxidase in Pepper under LL Stress

**Figure 7:**
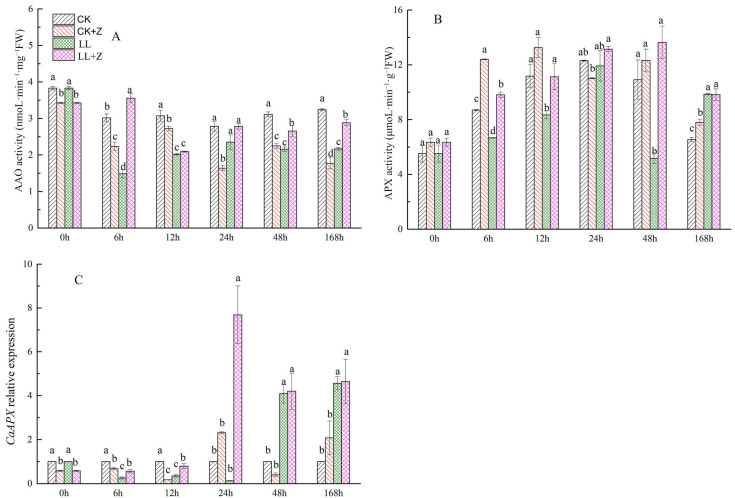
Effects of exogenous zeaxanthin on the activities of AAO and APX and *CaAPX* gene expression in pepper leaves under low temperature combined with low-light stress. (**A**) AAO activity; (**B**) APX activity; (**C**) *CaAPX* relative expression. CK: the pepper was sprayed with distilled water under normal conditions, CK + Z: the pepper was sprayed with zeaxanthin under normal conditions, LL: the pepper was sprayed with distilled water under low temperature combined with low-light stress, and LL + Z: the pepper was sprayed with zeaxanthin under low temperature combined with low-light stress. The values were presented as means ± standard deviations (SDs; *n* = 3). The different letters above bars indicated significant difference at *p* < 0.05 in the same period among treatments.

The results of our experiment showed that the AAO activity of pepper plants decreased under LL stress (Figure 7A). At 6 h after LL stress, the AAO activity was 1.49 nmoL·min^−1^·mg^−1^ FW. From 12 h after LL stress, there was a visible increase in AAO activity, but it was lower than that of CK, decreasing by 34.39%, 15.42%, 30.63%, and 33.14% from 12 to 168 h, respectively. However, in the case of LL + Z seedlings, there was an increase in AAO activity by 22.92% and 32.89% at 48 and 168 h, respectively, when compared with that of LL seedlings.

As shown in Figure 7B, the APX activity of pepper plants decreased during the initial stages of LL stress, but the activity was higher than that in CK seedlings after 168 h. However, LL + Z seedlings showed a significant increase in APX activity, but no difference was observed at 24 and 168 h between APX activities of LL and LL + Z seedlings. The expression of *CaAPX* was downregulated in the early stress phase and upregulated at 48 and 168 h with the increase in stress duration (Figure 7C). After exogenous Z treatment in the case of LL + Z seedlings, *CaAPX* gene expression significantly increased at 48 and 168 h when compared with that of LL seedlings.

### 3.10. Effects of Exogenous Zeaxanthin on Activities of Mono Dehydroascorbic Acid Reductase and Dehydroascorbic Acid Reductase and Their Gene Expression Levels in Pepper under LL Stress

**Figure 8:**
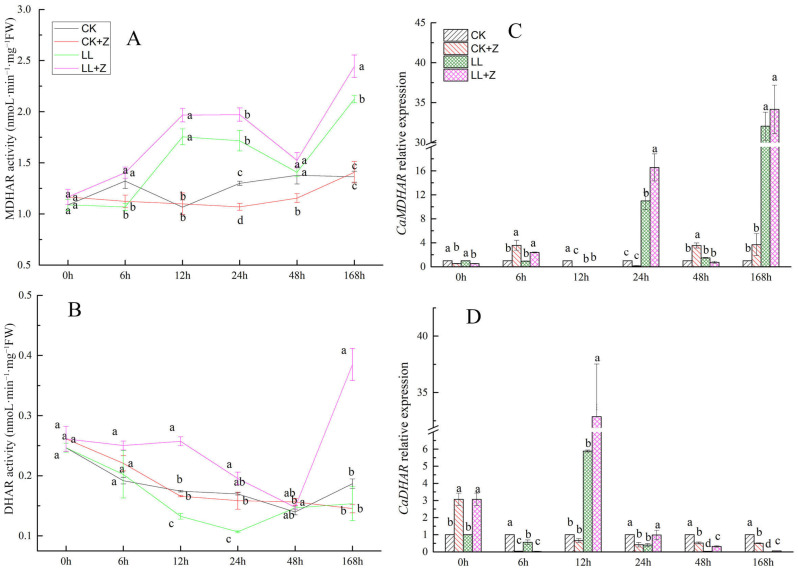
Effects of exogenous zeaxanthin on the expression levels of MDHAR, DHAR and related genes in pepper leaves under low temperature combined with low-light stress. (**A**) MDHAR activity; (**B**) DHAR activity; (**C**) *CaMDHAR* relative expression; (**D**) *CaDHAR* relative expression. The values were presented as means ± standard deviations (SDs; *n* = 3). CK: the pepper was sprayed with distilled water under normal conditions, CK + Z: the pepper was sprayed with zeaxanthin under normal conditions, LL: the pepper was sprayed with distilled water under low temperature combined with low-light stress, and LL + Z: the pepper was sprayed with zeaxanthin under low temperature combined with low-light stress. The different letters represent significant difference at *p* < 0.05 in the same period among treatments.

Figure 8A,B shows changes in the activities of two other key enzymes involved in the AsA–GSH pathway, namely MDHAR and DHAR. MDHAR activity of pepper seedlings treated with LL and LL + Z increased compared with that of CK seedlings (Figure 8A). LL stress caused a 55.66% increase in MDHAR activity after 168 h when compared with that of CK seedlings; In the case of LL + Z seedlings after 168 h, the increase in MDHAR activity was 15.07% higher than that of the LL seedlings. However, under normal conditions, CK + Z treatment did not show any effect on MDHAR activity. There was a significant reduction in DHAR activity in the case of LL seedlings at 12 h and 24 h, whereas LL + Z seedlings showed a significant increase in DHAR activity (Figure 8B).

Figure 8C,D shows the relative expression levels of the *CaDHAR* and *CaMDHAR* genes. Under LL stress, the *CaDHAR* gene was significantly downregulated compared with that under CK at all time points except at 12 h. Compared with that LL seedlings, in the case of LL + Z *CaDHAR* gene was significantly downregulated at 6 h after treatment, but in the subsequent time points, the expression of *CaDHAR* genes was significantly upregulated. *CaMDHAR* gene was downregulated under 12 h of LL stress, but it was upregulated after 24 h. *CaMDHAR* gene was significantly upregulated at 6 and 12 h under LL + Z treatment compared with that LL treatment. These results indicate that exogenous application of Z can enhance the expression of *CaDHAR* and *CaMDHAR* in plants, promote the AsA–GSH redox cycle, and increase the AsA content in vivo, thereby increasing the tolerance of pepper to LL stress.

## 4. Discussion

Low temperature combined with low light (LL) is complex abiotic stress that affects plant biochemistry through photoinhibition and oxidative stress [25,39]. Morphological changes were found in plants when suddenly exposed to unfavorable conditions compared to normal growth. Therefore, morphological changes may directly reflect crop tolerance to LL stress. In this study, LL stress significantly reduced plant height, stem diameter, and biomass (Table 2). However, the exogenous application of Z significantly improved the shoot biomass of seedlings. Our results are similar to those of previous studies where shoot dry weight of pepper seedlings with brassinosteroid application increased by 87.6%, compared to the control under the same LL stress conditions of 15/5 °C, 100 μmol m^−2^ s^−1^ [10]. In addition, RWC and EL can be used as important leaf indicators for measuring the water content and cell membrane damage in plant tissues [38,40,41]. Plants exposed to low temperatures show a reduction in leaf RWC and an increase in EL [42,43]. This study found that EL significantly increased in pepper seedlings under LL stress, and the RWC of pepper leaves initially decreased and then increased with increasing stress duration. However, the application of Z allowed the mitigation of LL stressed pepper seedlings where the seedlings showed reduced EL and improved RWC (Figure 1A,B); similar research on peppers supports this assumption [39]. This implies that Z may protect cell membrane integrity by increasing the number of unsaturated bonds in membrane lipids. Meanwhile, the higher RWC in leaves treated under LL may contribute to improving water absorption capacity and transpiration pull of roots caused by Z [43]. Moreover, the relative water content of mung bean seedling leaves was increased by increasing proline content after spraying spermidine [43], which was consistent with the changes in the RWC of leaves after spraying exogenous zeaxanthin in this study. It is suggested that exogenous zeaxanthin may improve the tolerance of seedlings to LL stress by increasing RWC caused by increasing proline content.

It has been shown that stomatal closure triggered by low temperatures can lead to a decline in photosynthetic capacity [44]. The decline in the photosynthetic performance of plants is attributed to stress conditions that include both stomatal and non-stomatal factors [45,46]. This study showed a significant reduction in Pn and Gs and an increase in Ci in the seedlings under LL stress (Table 3). This suggests the need for further studies to determine the reasons for the decrease in photosynthetic rate in pepper plants under LL stress. LL stress resulted in the closure of leaf stomata—which could be due to water loss from the leaves under stress conditions—leading to the shrinkage of guard cells. In addition, the RWC of the leaves was the same as that of the stomatal opening (24 h). However, the present study found that pre-treatment with exogenous Z could increase the net photosynthetic rate of pepper under LL stress conditions. This would further increase the accumulation of photosynthates to provide energy and material basis for normal growth of pepper plants. In this study suggest a close link between Z and LL resistance.

In plants, the chloroplast is the major site of ROS production, with H_2_O_2_ and O_2_^•−^ as the main ROS constituents [47]. ROS formation is triggered by abiotic stress [48]. LL stress not only affects plant growth and development but also causes excessive accumulation of ROS, leading to oxidative damage and eventually death of the plant tissue under severe conditions [49]. Therefore, the scavenging of ROS generated by abiotic stress is essential for plant growth and development. Our results show that pepper seedlings treated with Z under LL stress conditions (LL + Z) significantly increased the activities of antioxidant enzymes SOD (Figure 4A) and increased the content of antioxidants AsA and GSH (Figure 5A and Figure 6A). Moreover, quantitative analysis and histochemical detection of H_2_O_2_ and O_2_^•−^ showed that exogenous Z played an important role in preventing the accumulation of excessive ROS under LL stress (Figure 3). These observations suggested that Z might protect pepper seedlings from oxidative damage induced by LL stress. A similar study on pepper supports the observations made in this study [39]. This result may be due to the increased activity of antioxidant enzymes caused by the upregulated expression of *CaSOD* and *CaCAT* genes, effectively scavenging H_2_O_2_ and O_2_^•−^. Consistent with this, certain studies have shown that exogenous brassinosteroids alleviate cold-stress-induced damage by enhancing the antioxidant enzyme activity of pepper [10]. However, when compared with that of LL, the POD activity of LL + Z decreased, and the expression of the *CaPOD* gene was downregulated (Figure 4B,E). These results differ from the current findings, and this discrepancy may be due to the different species tested and the different treatment conditions.

In higher plants, the AsA–GSH circulation system is an integral part of the antioxidant system and a major pathway for H_2_O_2_ [50]. Additionally, AsA–GSH pathway can cooperate with antioxidant enzymes and help in efficient ROS scavenging [51], especially when plants experience abiotic stress [52]. AsA and GSH are the major antioxidant substances in the AsA–GSH cycling system that can resist oxidative stress caused by ROS in plant cells and maintain the stability of the antioxidant system in vivo [53,54]. DHA and GSSG are the oxidizing substances of AsA and GSH, respectively; AsA/DHA and GSH/GSSG ratios may reflect the redox capacity of plants. In general, the higher the content of reducing substances, the stronger the stress resistance of plants [55,56]. This study showed an increase in AsA and GSH content in pepper seedlings pretreated with Z and subjected to LL stress (Figure 5A and Figure 6A). The ratios of AsA/DHA and GSH/GSSG also increased under Z pre-treatment, which contributed to the reduction of oxidative stress as indicated by reduced H_2_O_2_ and O_2_^•−^ levels. This could be attributed to an increase in DHAR activity and *CaDHAR* expression. Consistent with the results of previous studies, the AsA/DHA ratio increased in transgenic tobacco overexpressing *DHAR*, resulting in increased tolerance to NaCl stress [57]. These results were confirmed by a previous study. Similar results have been reported in tomatoes, where exogenous ALA at appropriate concentrations has increased the ratio of AsA/DHA to GSH/GSSH, which improved the antioxidant capacity of tomato plants at low temperatures [58]. High levels of AsA and GSSH have increased the antioxidant capacity of the AsA–GSH cycling system and improved the cold tolerance of pepper fruits [59]. These results suggest that Z treatment can regulate the redox status of AsA and GSH in cells, maintain high AsA/DHA and GSH/GSSH ratios, accelerate ROS degradation, and maintain membrane integrity, consequently improving the tolerance of pepper seedlings to LL.

AAO is a glycoprotein localized in the plant cell wall that can directly oxidize AsA. This experiment indicates that exogenous Z increases AAO activity in pepper leaves under LL stress conditions (Figure 7A). A similar study of the effects of exogenous 5-aminolevulinic acid (ALA) on cucumber seedlings under salt stress found that exogenous ALA could enhance AAO activity of cucumber leaves under salt stress [60]. Overexpression of AAO in tobacco plants completely oxidized AsA in the symplast to DHA but induced a reduction in the redox state of AsA in the apoplast. This led to an increase in GSH and a decrease in GSSG contents, indicating an increase in GSH/GSSG ratio [61]. In addition, plants overexpressing AAO exhibit enhanced stem growth more than wild-type plants [62]. In the present study, the application of Z under LL stress increased the AAO activity of peppers, confirming that higher AAO content in leaves could improve the plant height of peppers by regulating the value of the oxidation/reduction ratio of the AsA pool, thus alleviating oxidative stress caused by LL.

In plant cells, DHAR is detected in the cytoplasm, mitochondria, and chloroplasts. DHAR catalyzes GSH reduction from DHA to generate AsA and GSSG and regulates the AsA/DHA ratio. MDHAR catalyzes the reduction of MDHA to generate AsA, which ensures the efficient regeneration of AsA [55]. Increased activity of APX balances the AsA–GSH reaction pool, which helps maintain redox homeostasis and reduce oxidative damage to cells [63]. In the present study, MDHAR and DHAR activity under LL stress increased significantly with the increasing LL stress duration. Exogenous Z significantly increased the activity of MDHAR and DHAR under stress and improved their ability to intercept ROS. In addition, genes involved in the AsA–GSH cycle (*CaAPX*, *CaDHAR,* and *CaMDHAR*) were upregulated by Z treatment under stress conditions. This result is concurrent with the results obtained by Yao et al. (2021) [64] in the case of pepper fruit treated with exogenous GSH; GSH treatment in pepper fruits can increase the activities of APX, DHAR, and MDHAR enzymes and induce the expression of related genes, thereby improving cold resistance and preventing cold-induced damage in pepper fruits. These results also confirmed that Z has a positive effect on the regulation of the AsA–GSH cycle under LL stress. In addition, Z pre-treatment increased AsA and GSH levels thereby improving the circulation efficiency of AsA–GSH (Figure 5A and Figure 6A). This has further enhanced the ability of pepper plants to remove excess ROS thus improving their tolerance to LL stress.

## 5. Conclusions

The results of this study suggest that foliar spray of pepper leaves with Z before LL stress can promote seedling growth, reduce membrane electrolyte loss, increase the photosynthetic rate, and induce stomatal opening. Z pre-treatment caused the upregulation of *CaSOD*, *CaCAT,* and the subsequent increase with respect to the enzyme activities of SOD and CAT in pepper seedlings was increased. Z pre-treatment also increased the expression of key enzymes and related genes (*CaAPX*, *CaMDHAR*, and *CaDHAR*) in the AsA–GSH cycle, leading to a significant increase in the content of antioxidant substances (AsA and DHA). After pretreatment with Z, the content of H_2_O_2_ and O_2_^•−^ under LL stress decreased significantly, which was due to the increased activity of antioxidant enzymes and the high content of antioxidant substances. Therefore, this study concludes that pre-treatment with Z under LL conditions can mitigate LL damage to pepper seedlings to a significant extent. The improved tolerance of pepper seedlings to LL stress in this study provides a certain theoretical basis for future studies involving the resistance of peppers to abiotic stress.

## Figures and Tables

**Table 1:** The sequences of primers used for the qRT-PCR.

Gene Name	Sequence (5′–3′)	GenBank Accession Number
*CaSOD*	F:GTGAGCCTCCAAAGGGTTCTCTTG	AF036936.2:35–721
	R: AAACCAAGCCACACCCAACCAG	
*CaPOD*	F: GCCAGGACAGCAAGCCAAGG	FJ596178.1:68–1042
	R: TGAGCACCTGATAAGGCAACCATG	
*CaCAT*	F: TTAACGCTCCCAAGTGTGCTCATC	NM_001324674.1:72–1550
	R: GGCAGGACGACAAGGATCAAACC	
*CaAPX*	F: TGTTGTTGCTGTTGAGGTCACTGG	AF442387.1:24–887
	R: CATCTGGTAACCGCCCTTCCTTTG	
*CaDHAR*	F: CCATATGTCAAAGGGCAGAA	KJ950368.1:1:64-702
	R: CTTTCAGGCACACTCCACTT	
*CaMDHAR*	F: TACTTCTACTCCCGTGCCTT	XM_016687442.1:79-1380
	R: GAGGAATGCACCAACGATCT	
*Actin*	F: GTCCTTCCATCGTCCACAGG	XM_016722297.1
	R: GAAGGGCAAAGGTTCACAACA	

Note: F: Forward primer; R: Reverse primer.

**Table 2:** Effects of exogenous zeaxanthin on growth indexes of pepper under low temperature combined with low-light stress.

Treatment	Height (cm)	Stem (mm)	Fresh Weight of Shoot (g)	Fresh Weight of Root (g)	Dry Weight of Shoot (g)	Dry Weight of Root (g)
CK	16.18 ± 0.299 a	2.86 ± 0.111 a	5.08 ± 0.153 a	2.02 ± 0.121 a	0.444 ± 0.018 a	0.104 ± 0.005 a
CK + Z	16.30 ± 0.564 a	2.82 ± 0.136 a	5.39 ± 0.372 a	2.06 ± 0.107 a	0.443 ± 0.025 a	0.111 ± 0.011 a
LL	12.46 ± 0.289 c	2.39 ± 0.081 b	3.50 ± 0.083 b	1.20 ± 0.068 b	0.359 ± 0.008 b	0.073 ± 0.004 b
LL + Z	13.91 ± 0.209 b	2.72 ± 0.096 a	3.93 ± 0.068 b	1.31 ± 0.086 b	0.418 ± 0.012 a	0.076 ± 0.001 b

Note: The values were presented as means ± standard deviations (SDs; *n* = 5); different lowercase letters in the same column indicate significant differences at *p* < 0.05 level.

**Table 3:** Effects of exogenous zeaxanthin on photosynthetic parameters of pepper under low temperature combined with low-light stress.

Treatment	Pn (μmol m^−2^ s^−1^)	Ci (μmol m^−2^ s^−1^)	Tr (mmol m^−2^ s^−1^)	Gs (mmol m^−2^ s^−1^)
CK	14.133 ± 0.285 a	348.000 ± 4.360 b	3.300 ± 0.058 a	112.667 ± 6.360 a
CK + Z	12.067 ± 1.396 a	375.333 ± 19.471 b	2.533 ± 0.176 b	96.000 ± 9.019 a
LL	0.267 ± 0.067 c	479.667 ± 5.175 a	1.167 ± 0.090 c	40.133 ± 2.862 c
LL + Z	5.000 ± 0.153 b	343.667 ± 12.170 b	1.367 ± 0.033 c	69.667 ± 2.028 b

Note: The values were presented as means ± standard deviations (SDs; *n* = 3); different lowercase letters in the same column indicate significant differences at *p* < 0.05 level.

## Data Availability

The data that support the findings of this study are available on request.
